# Supplementary material for: CRISPR-Cas9 Mediated Knockout of SagD Gene for Overexpression of Streptokinase in Streptococcus equisimilis
Source: Microorganisms. 2022 Mar 17;10(3):635. doi: 10.3390/microorganisms10030635 (PMC8953821; doi:10.3390/microorganisms10030635)
Supplement: Supplementary file 1 [file microorganisms-10-00635-s001.zip › microorganisms-1581118-supplementary.pdf]

microorganisms

Supplementary materials

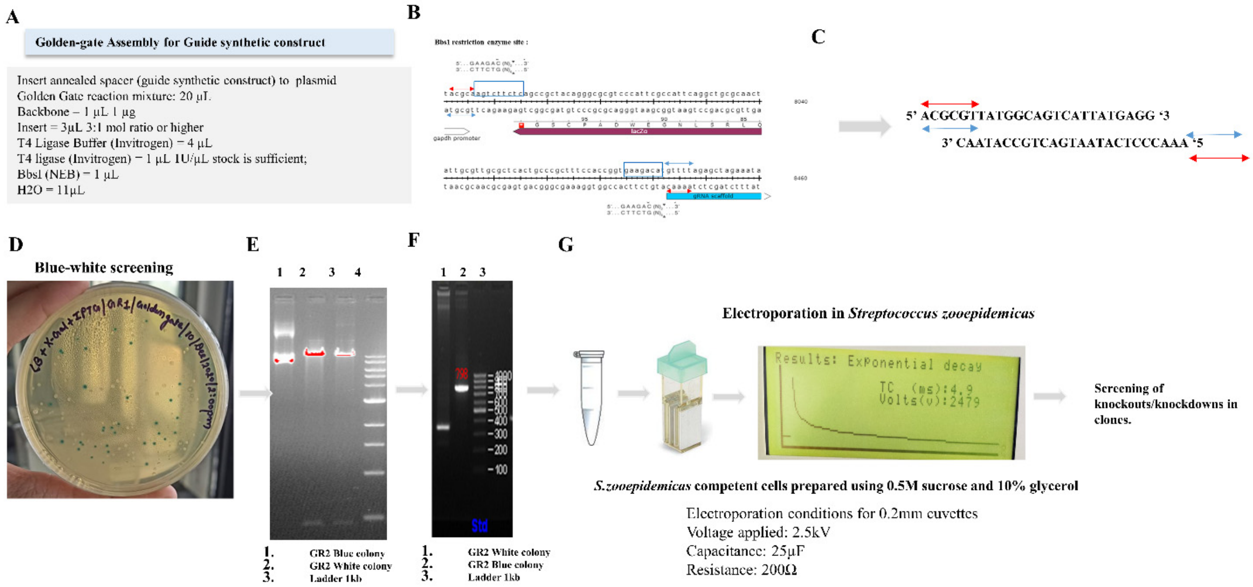

Figure S1. Golden gate assembly protocol for chimeric plasmid preparations. **(A)** Golden gate reaction mixture where BbsI and T4-DNA ligase were added into a single tube and PCR conditions favoring simultaneous cut and ligation were applied**. (B)** Two sites of BbsI were present in pCRISPomyces-2 plasmid to produce staggered overhangs. **(C)** Guide RNA sequence**s** with ACGC and CAAA overhang were designed. These overhangs were complementary to BbsI restriction sites for better ligation. **(D)** After heat-shock transformation of the chimeric pCRISPomyces-2 plasmid into *E.coli* top 10F′ cells, blue-white screening was used to select the plasmid containing guide RNA construct. **(E)** White transformation was further validated using BbsI restriction where the blue colony will yield a single 10 kb product and Transformants (white colony) will yield intact supercoiled, nicked plasmid as they didn't possess BbsI restriction site anymore. **(F)** clones were also validated using pCRISPmyces-2 F & R primers, where blue colony having intact LacZ gene will yield ~800bp band and the white colony will yield only ~300 bp due to deletion of lacZ gene showing guide RNA insertion. **(G)** *Streptococcus equisimilis* was washed with sucrose and 10% (*v*/*v*) glycerol and further transformed using 0.2 mm electroporation cuvettes of Biorad gene pulser.

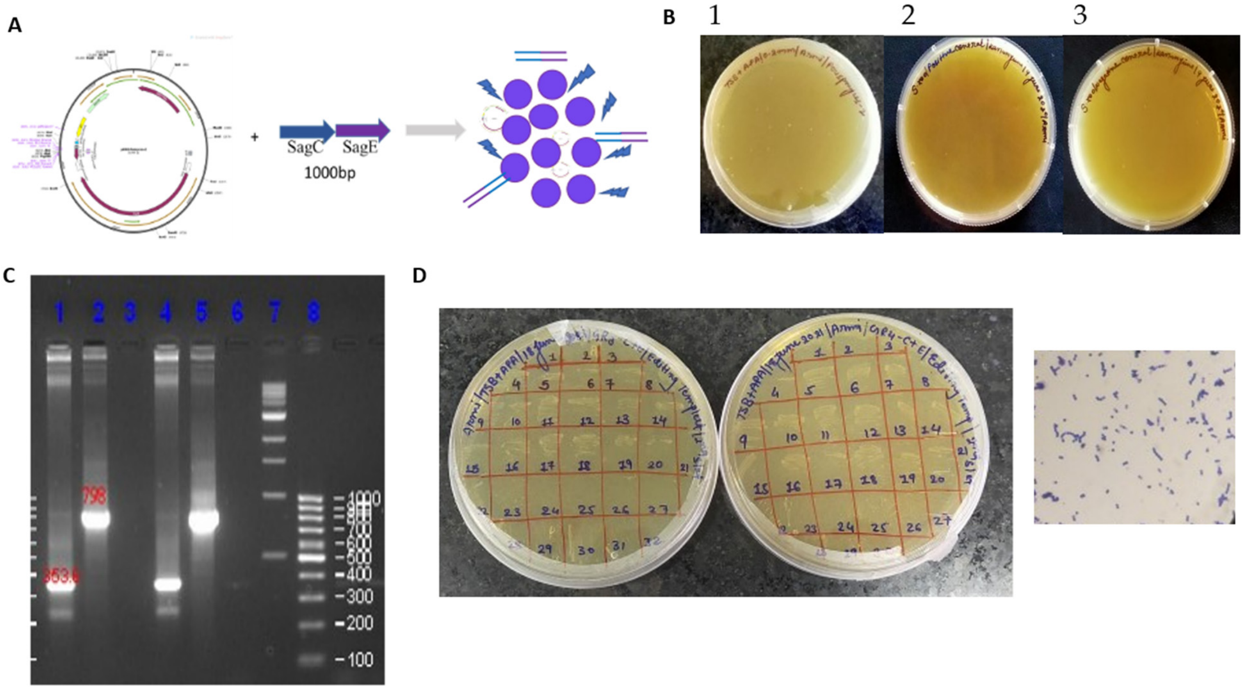

Figure S2. PCRISPomyces-2 insertion in host **(A)** Schematic representation of pCRISPomyces-2 plasmid and strategy for electroporation *Streptococcus equisimilis* cells. **(B)** transformation of pCRISPomyces-2 into *Streptococcus equisimilis* using electroporation. **(B1)** GR3 transformants **(B2)** Positive control **(B3)** Negative control. **(C)** PCR amplification for confirmation of pCRISPomyces-2 plasmid using pCRISPomyces2_GR. Control cells have intact lacZ gene so amplification product is 800bp and GR3 clones with deletion of LacZ gene showed around 350 bp amplification. **(D)** Random colonies were picked up and transferred in to TSB + Apramycin containing medium. Gram staining was always performed to confirm purity of culture.

For expression of guide RNA cassette, *S. equisimilis* clones with chimeric pCRISPomyces-2 plasmids bearing SagD gene-specific gRNA constructs were grown for 2-3 days at 28 °C in TSB medium with 50 µg/L Apramycin. However, we were unable to achieve gene editing with these two gRNA cassettes, GR1 and 2. The sequencing results always followed a single pattern in which the region matching to gRNA showed mixed peaks whereas the outside region (other than the 20bp guide RNA) had excellent coverage during DNA sequencing. This prompts us to look into the sequencing results in further depth. We grew the clones at two different temperatures: 28 °C and 37 °C. Because 28 °C is an appropriate temperature for pCRISPOmyces-2 expression (plasmid curation occurs at 37 °C), continuous expression (at lower levels) of CRISPR array may exist in organisms, resulting in the generation of diverse strains with gene-edited and unedited regions. As a result, during sequencing, mix peaks comprising chromatograms were obtained **(Figure 1G,H).** The poly peak parser helped in identifying the second significant sequence in the chromatogram from the region covering the SagD gene's gRNA. Our analysis revealed that these second significant sequences lacked a 21 bp gRNA complementary region, as indicated by the purple arrow in **Figure 1F.** These sequencing data leads us to believe that there is an issue in the expression of higher transcripts of gRNA to bind specific genomic regions (*SagD*) for endonuclease activity **(Figure 1F)**. To overcome this issue, other gRNAs sequences were designed to have an efficient match up to 15-16 nucleotides out of 24 to *SagD* gene with a change in plasmid expression conditions have been shown in the material and method section.

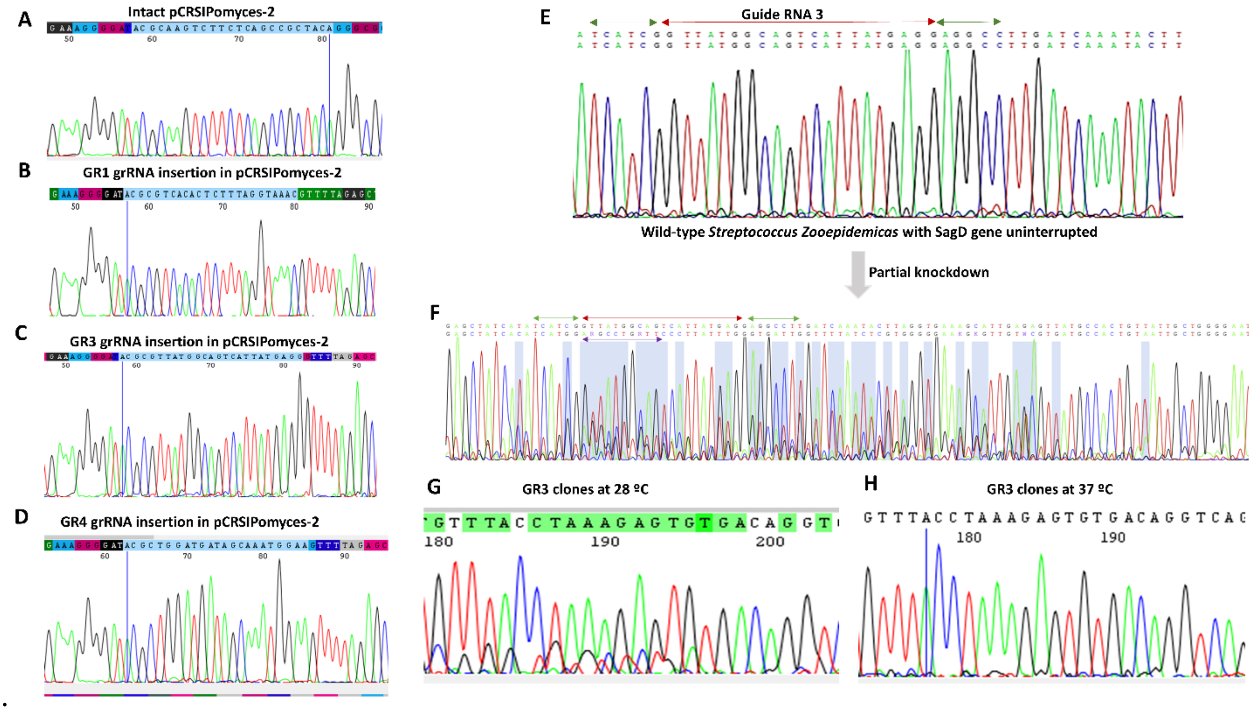

Figure S3. CRISPR-Cas9 system for knockout. **(A)** Intact pCRISPomyces-2 plasmid. **(B)** Incorporation of GR2 gRNA sequence into PCRISPomyces-2 plasmid. **(C)** Incorporation of GR3 gRNA sequence into PCRISPomyces-2 plasmid. **(D)** Incorporation of GR4 gRNA sequence into PCRISPomyces-2 plasmid. **(E)** Native *sagD* gene sequence having intact/uninterrupted GR3 sequence. Red arrow showed the region covering GR3, green arrows were overlapped regions covering GR3. **(F)** Poly-peak parser output for the clone sequences contained mixed peaks for *sagD* gene. Purple arrow shows the sequence similar to the green arrow next to the GR3 sequence conforming mix population of clones having both edited and unedited *sagD* gene. **(G)** pCRISPomyces-2 clones grown at 28 °C. **(H)** pCRISPomyces-2 was temperature-sensitive plasmids due to the present of PGF5. If clones having CRISPomyces-2 were grown at 37 °C they have curated the plasmid. If clones were grown at 28 °C they express the pCRISPmyces 2 plasmids and editing might occur. At 37 °C, no mix peaks were observed while at 28 °C, same sequence were there but mix peaks were observed. This may be possible because of the low expression of pCRISPomyces 2 plasmids for a complete knockout, which is leading to a mixing population having edited and unedited *sagD* gene.

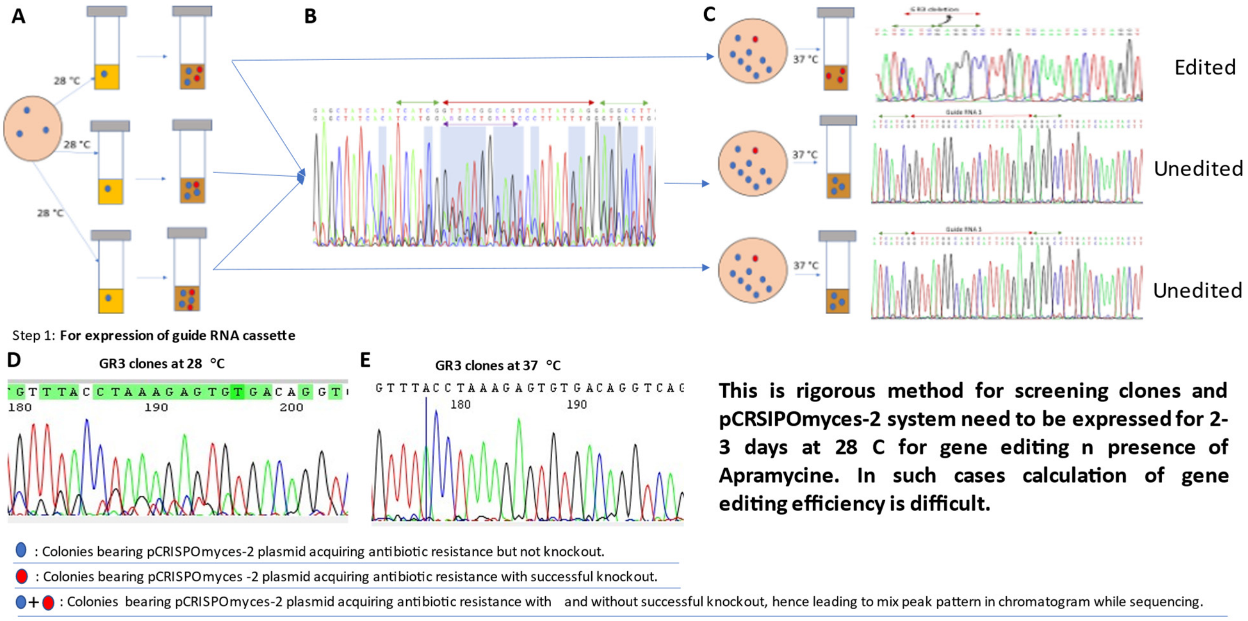

Figure S4. Screening and identification of knockout clone: (A) Schematic representation of colony picking and growing in media containing TSB + apramycin for expression of guide RNA and Cas9 cassette. **(B)** Colonies bearing pCRISPOmyces-2 plasmid acquiring antibiotic resistance with and without successful knockout, hence leading to mix peak pattern in chromatogram while sequencing. **(C)** To overcome issue of mix culture of wild-types and mutants, tubes having bacterial growth in apramycin were streaked into tsb+apramycine media. Future random colonies were picked up having change of mutant and further growth to 37 for stopping expression of cas9 cassette, hence leading to successful separation of mutant and wild-type. **(D)** To prove further that this cassette is continuously expressing at 28C and 37 C both, we further performed experiment where single colony of clones is subjected to TSB at 28 ºC and 37 ºC both and chromatogram. **(E)** pCRISPomyces-2 clones grown at 37 °C

Such kind of rigorous method is developed for identifying edited from edited. In this case we got low gene editing efficiency **Gene editing efficiency = expected sequence counts/total counts*100%**, 4.44%, however it is not our primary concern. SagD gene abruption is our major focus, which is achieved as results.

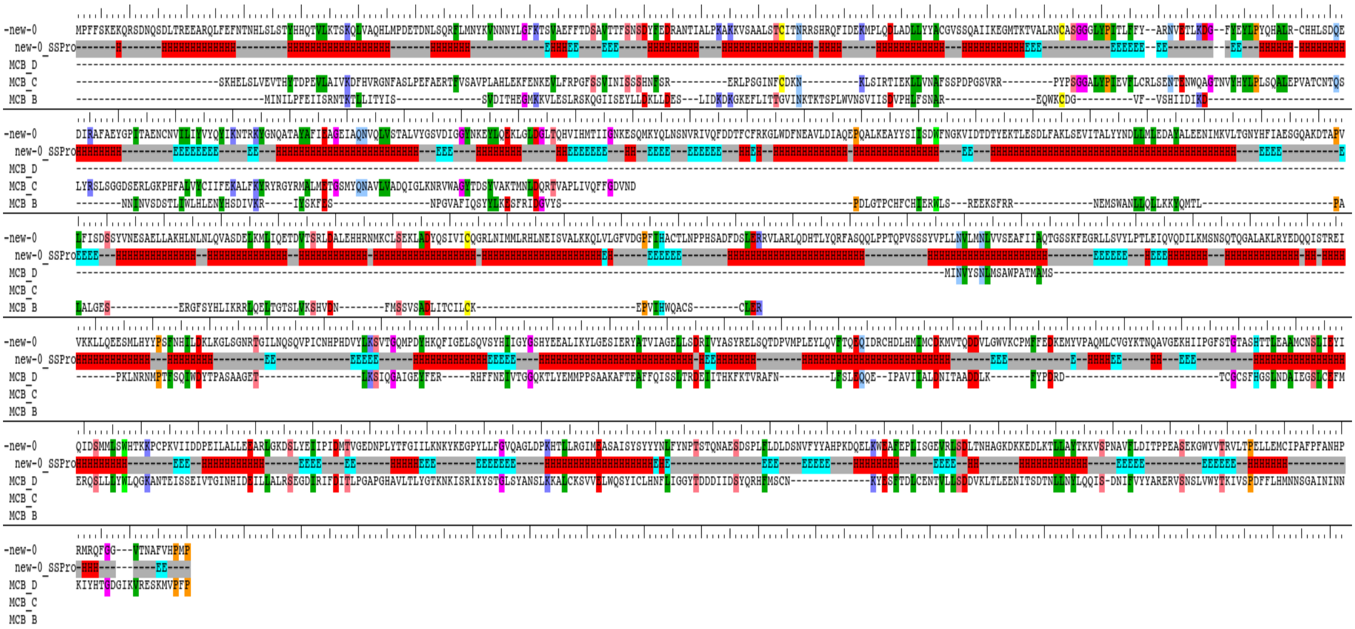

Figure S5. Sequence alignment of SagBCD complex with wild-type *E.coli* MCB complex is shown here. Red colored region is alpha helix and blue colored region is beta sheets.

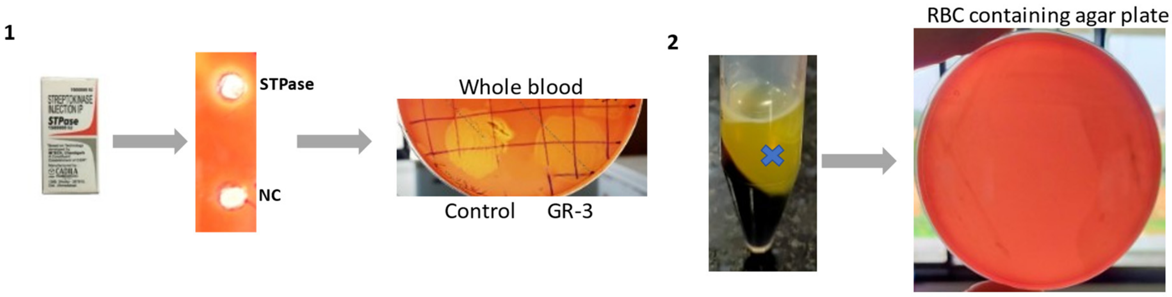

Figure S6. (1) Streptokinase standard is available as STPase, produced by Cadila pharmaceuticals. STPase gave partial zone on blood agar plate, reason behind it was presence of euglobin fraction in blood. Euglobin is substrate for streptokinase. Hence partial zone was observed. Streptokinase activity is measured by plasma faction and streptolysin activity is measured by erythrocytes. To separates substrate for both factors blood was subjected to centrifugation at 4000 rpm for 30 mins to

separate out both sections. **(2)** Erythrocytes were dissolved in PBS and used in RBCs agar plate formation. GR-3 clones were showing no zone of hemolysis in RBCs agar plate, which supports the altered streptolysine activity.

Table S1. List of all the organisms used in the study.

Sr no.	Name of the Organism	Selection
1.	*E. coli Top10F'*	NA
2.	*E. coli Top10F'*-pCRISPomyces2	Apramycin
3.	*E. coli Top10F'*-pCRISPomyces2-GR1	Apramycin
4.	*E. coli Top10F'*-pCRISPomyces2-GR2	Apramycin
5.	*E. coli Top10F'*-pCRISPomyces2-GR3	Apramycin
6.	*E. coli Top10F'*-pCRISPomyces2-GR4	Apramycin
7.	*S. equimilus*	NA
8.	*S. equimilus* -pCRISPomyces2	Apramycin
9.	*S. equimilus* -pCRISPomyces2-GR1	Apramycin
10.	*S. equimilus* - pCRISPomyces2-GR2	Apramycin
11.	*Streptococcus equimilus* - pCRISPomyces2-GR3	Apramycin
12.	*Streptococcus equimilus* - pCRISPomyces2-GR4	Apramycin

Table S2. List of primer and gRNAs sequences used in this study.

Sr no.	Name of the Primer Pairs	Forward Sequence(5′-3′)	Reverse Sequence(5′-3′)
1.	SagD gene	ACAAGGGGCTCTGGCAAAAT	AAGAGCACAGCCAAGCTCAA
2.	Streptokinase gene	ATGTTGACTTCAAAAAGAC	TTATTTTTGCAGGTCTT
3.	pCRISPomyces2_GR	ACGGCTGCCAGATAAGGCTT	TTCGCCACCTCTGACTTGAG
4.	SKC_RT	ATACATCTTGACGGGTCAGG	AAGAGACCCTGCTGCCAT
5.	SagD_RT	GACAGCCTCTCATACAACAC	AGCGGATTATCCTCTCCAAC
6.	GR1_SagD	ACGCGTCACAC-TCTTTAGGTAAAC	AAACGTTTACCTAAAGAGTGTGAC
7.	GR2_SagD	ACGCGGACTTAGTGGTAAC-CGAAC	AAACGTTCGGTTACCACTAAGTCC
8.	GR3_SagD	ACGCGTTATGGCAG-TCATTATGAGG	AAACCCTCATAATGACTGCCATAAC
9.	GR4_SagD	ACGCTGGATGATTAG-CAAATGGAA	AAACTTCCATTTGCTAATCATCCA
